# IL33-induced neutrophil extracellular traps (NETs) mediate a positive feedback loop for synovial inflammation and NET amplification in rheumatoid arthritis

**DOI:** 10.1038/s12276-024-01351-7

**Published:** 2024-12-02

**Authors:** Jifeng Tang, Jinfang Xia, Huali Gao, Renquan Jiang, Lianbo Xiao, Huiming Sheng, Jinpiao Lin

**Affiliations:** 1 Department of Laboratory Medicine, Tongren Hospital, Shanghai Jiao Tong University School of Medicine, Shanghai, China; 2 Department of Orthopedic Surgery, Guanghua Hospital Affiliated to Shanghai University of Traditional Chinese Medicine, Shanghai, China; 3 Institute of Arthritis Research in Integrative Medicine, Shanghai Academy of Traditional Chinese Medicine, Shanghai, China; 4 Department of Laboratory Medicine, The First Affiliated Hospital of Fujian Medical University, Fuzhou, China; 5 Faculty of Medical Laboratory Science, College of Health Science and Technology, Shanghai Jiao Tong University School of Medicine, Shanghai, China

**Keywords:** Autoimmunity, Rheumatoid arthritis

## Abstract

This study investigated the mechanisms driving the induction and sustained presence of neutrophil extracellular traps (NETs) in the synovial microenvironment of rheumatoid arthritis (RA). Synovial tissue and fluid samples were collected from patients with RA and osteoarthritis (OA), and NET levels and cytokine concentrations were measured using a cytometric bead array and enzyme-linked immunosorbent assay (ELISA). The ability of interleukin-33 (IL-33) to induce NET formation was evaluated using quantitative assays, immunofluorescence staining, live-cell imaging, and electron microscopy. Coincubation experiments of NETs with fibroblast-like synovial cells (FLSs) were conducted, and a modified Transwell migration assay was designed to assess neutrophil migration. The role of IL-33 and NETs in RA progression was further investigated using a collagen antibody-induced arthritis (CAIA) mouse model. The results revealed an increase in NETs and IL-33 levels in the synovial fluid of RA patients, with a significant positive correlation between them. NET formation assays confirmed that IL-33 activates neutrophils to produce NETs and that neutrophils from RA patients exhibit increased responsiveness to IL-33 stimulation. Both in vitro and in vivo evidence has demonstrated that NETs stimulate FLSs to secrete IL-33 and the chemokine CXCL8 via Toll-like receptor 9, promoting further neutrophil recruitment and increasing NET production within the RA synovium. This study reveals a novel positive feedback loop involving NETs and FLSs that is mediated by IL-33 that increases NET accumulation in RA. Targeting IL-33 or NET formation and amplification may offer new therapeutic strategies for managing RA.

## IL-33 drives neutrophil extracellular traps in rheumatoid arthritis

Rheumatoid arthritis is a disease where the body’s defense system wrongly attacks the joints, leading to swelling and pain. This research explores the role of neutrophil extracellular traps in RA. NETs are net-like structures released by a type of white blood cell to catch harmful germs but in RA, NETs can harm the body’s own tissues. The team collected samples from 120 RA patients, comparing them with samples from osteoarthritis patients and healthy people. They studied how certain proteins, particularly IL-33, affect NET formation in RA patients’ joints. They found that IL-33 greatly increases NET production, suggesting a cycle where IL-33 and NETs continue the inflammation in RA. This highlights IL-33’s potential as a target for RA treatment, providing new understanding of this disabling disease. Future treatments might aim to break this cycle to ease RA symptoms.

This summary was initially drafted using artificial intelligence, then revised and fact-checked by the author.

## Introduction

Rheumatoid arthritis (RA) is a chronic inflammatory autoimmune disease that primarily affects joints and is often accompanied by extra-articular manifestations.^1^ The development of RA is influenced by various genetic, epigenetic, and environmental factors.^2,3^ Despite extensive research, the exact etiology and pathogenesis of RA remain incompletely understood, and RA remains clinically incurable.^4^

Although the precise etiology remains to be established, it is thought that RA results from a breach in immune tolerance that is characterized by an increase in the activity of autoreactive immune cells and the production of autoantibodies.^3,4^ Hypercitrullination stands out as a notable feature of RA. Anti-citrullinated protein antibodies (ACPAs), which target various citrullinated peptides and proteins specifically, are the most prevalent autoantibodies in RA patients and serve as highly specific serological biomarkers for RA.^5,6^ During RA progression, the abnormally accumulated citrullinated products in RA convert to self-antigens and further change to autoantigens, ultimately leading to RA symptoms.^5^ Neutrophils serve as a significant source of citrullinated autoantigens and peptidyl arginine deiminases (PADs), enzymes that catalyze the conversion of arginine to citrulline.^7^ Notably, neutrophils are plentiful in the inflamed joints of RA patients, particularly during the early stages of the disease.^8^ The abundance of extracellular citrullinated autoantigens and PADs in RA synovial fluid suggests a mechanism whereby intracellular PADs and citrullinated autoantigens are released into the extracellular space through neutrophil death in the RA synovial microenvironment. Therefore, it is unsurprising that neutrophil extracellular traps (NETs), which represent a novel and distinct form of neutrophil death, have garnered considerable attention in RA pathogenesis.

NETs are extracellular structures composed of nuclear and granule contents that can entrap and kill fungi, bacteria and viruses.^9^ Most often, NET formation can be triggered by various stimuli, including microorganisms, cytokines, immune complexes, autoantibodies, crystals, chemicals and platelets. This activation leads to the mobilization of the NADPH oxidase machinery and the subsequent generation of reactive oxygen species (ROS).^10^ Consequently, several proteases, notably neutrophil elastase (NE) and myeloperoxidase (MPO), are released, which degrade and cleave histones and structural proteins. Moreover, protein citrullination of histones by PAD4 promotes the decondensation of chromatin. As research advances, investigators have discovered that NETs can orchestrate complex physiological and pathological responses, contributing to tissue damage repair, immunoregulation, inflammation, tumor metastasis, and development.^11^ Abnormalities in NET formation and degradation can lead to a range of diseases, including thrombosis, organ dysfunction, and systemic autoimmune and inflammatory disorders.^10–12^

Recent discoveries have highlighted the central roles of NETs in the initiation and perpetuation of systemic autoimmune disorders. Many molecules released by neutrophils in NETs are autoantigens known to be targeted by the adaptive immune system in systemic autoimmunity.^10^ In RA, in addition to the release of many modified autoantigens and PADs, increased NET levels are associated with the levels of ACPAs and systemic inflammatory markers.^13,14^ Within the RA synovium, NETs mediate articular cartilage damage and increase the immunogenicity of cartilage components by facilitating their citrullination.^15^ It has also been suggested that NETs and carbamylated NETs instruct monocytes to undergo rapid osteoclast formation to promote bone erosion.^16^ Overall, neutrophils seem to play a central inflammatory role in RA and promote pathogenic adaptive immunity via NET formation. Although the roles of NETs have been reported in RA, the precise mechanisms underlying their induction and long-term maintenance in the RA synovial microenvironment still require further investigation. Hence, the aim of our current study was to explore the factors contributing to elevated NET levels in the RA synovium. Through our investigation, we revealed a mechanism indicative of a positive feedback loop that contributes to the amplification of NET levels within the RA synovium.

## Methods

### Patients and healthy donors

A total of 120 RA patients were recruited for the study, all of whom satisfied the 2010 American College of Rheumatology/European League Against Rheumatism (ACR/EULAR) classification criteria^17^ for RA. In addition, 82 osteoarthritis (OA) patients who fulfilled the 2018 ACR guidelines^18^ and 98 healthy donors (HDs) were included as controls. Peripheral blood samples were collected from each participant after a 12-h fast. RA disease activity was evaluated on the basis of the disease activity score of 28 joints (DAS28) score system, which was evaluated by a rheumatologist using the following formula:^19^ DAS28 = 0.56*sqrt(TJC) + 0.28*sqrt(SJC) + 0.36*Ln(CRP + 1) + 0.014*VAS + 0.96. Here, TJC = tender joint count, SJC = swollen joint count, and VAS = visual analog scale. Synovial tissues were obtained from patients undergoing routine synovectomy at Guanghua Hospital Affiliated to Shanghai University of Traditional Chinese Medicine, Shanghai, China. The study was approved by Tongren Hospital, Shanghai Jiao Tong University School of Medicine, Shanghai, China (ethical batch number: AF/SC-11/04.0), and the authors declare no violation of the Declaration of Helsinki on human experimentation. Verbal and written informed consent were obtained from all participants. The clinical characteristics of all the participants are summarized in Supplementary Table 1.

### Animals

All the animal experiments were conducted in accordance with the Institutional Animal Care and Use Committee guidelines. Six- to eight-week-old male DBA/1J mice were purchased from the Shanghai Laboratory Animal Center, Chinese Academy of Science. The mice were housed in a specific-pathogen-free (SPF)-grade animal facility and maintained under controlled conditions with a 12-h light/dark cycle. They had ad libitum access to food and water throughout the experiment.

### Quantification of NET–DNA complexes and cell-free DNA (cfDNA)

To quantify NETs in the cell culture supernatant, a Quant-iT PicoGreen dsDNA assay kit (Invitrogen, USA) was used following the manufacturer’s guidelines. Briefly, 2 μL of cell supernatant was diluted to 100 μL with TE buffer (10 mm Tris-HCl, 1 mm EDTA, pH 7.5) and added to each well. Then, a prepared working solution of PicoGreen reagent was added to each well, and the mixture was incubated for 5 min at room temperature in the dark. The DNA content was calibrated via the Qubit dsDNA HS Standard #1 and the Qubit dsDNA HS Standard #2 (Invitrogen). The fluorescence of the samples was measured using a Qubit 4 fluorometer (Thermo Fisher Scientific, USA) with excitation at 480 nm and emission at 520 nm. Similarly, for the measurement of cfDNA in peripheral serum or synovial fluid, 2 μL samples were diluted to 100 μL with TE buffer, and the subsequent steps were consistent with those described above.

### NET enzyme-linked immunosorbent assay (NET-ELISA)

To quantify NETs in peripheral serum or synovial fluid, a modified NET-ELISA method was used. Briefly, a 96-well immunoplate was coated with an anti-histone H3 (citrulline R17) capture antibody or an anti-myeloperoxidase (MPO) antibody and incubated overnight at 4 °C. The next day, the plate was washed with PBS/0.2% Tween-20 and blocked with PBS/5% BSA for 2 h at room temperature. Then, 100 μL of peripheral serum or synovial fluid was added to each well, and the samples were incubated for 90 min at 37 °C and then washed. Finally, the Quant-iT PicoGreen dsDNA assay kit (Invitrogen) was used according to the manufacturer’s instructions to quantify the DNA concentration with a Qubit 4 fluorometer (Thermo Fisher Scientific). All the antibodies used were commercially validated, and the antibody usage information is provided in Supplementary Table 2.

### Cytometric bead array and ELISA

A cytometric bead array (CBA) was used to quantify multiple human cytokines in human synovial fluid simultaneously, including TNF-α, IFN-γ, IL-1β, IL-2, IL-2R, IL-4, IL-5, IL-6, IL-8, IL-10, IL-17A, and IL-33, following the manufacturer’s instructions. All reagents used in this process have been commercialized, are used in clinics, and were procured from Qingdao Raisecare Biotechnology Co., Ltd. (China). Flow cytometry was conducted on a BriCyte E6 flow cytometer (Mindray, China), and the results were analyzed using MRFlow software (Mindray).

In subsequent experiments, to ensure sufficient quantification ranges, commercial ELISA kits for human IL-33, soluble stimulation 2 (ST2), and the CXCL8 Quantikine ELISA Kit (R&D Systems, USA) were used. All ELISAs were conducted following the manufacturer’s instructions.

### Human neutrophil isolation and stimulation and NET preparation

Human neutrophils were isolated from EDTA-anticoagulated peripheral blood via Percoll density gradient centrifugation (Solarbio, China). Neutrophil numbers and purity were confirmed using an auto hematology analyzer (BC-6800Plus, Mindray, China), and neutrophils with a purity exceeding 95% were used for subsequent experiments. Neutrophils (1 ×10^6^ cells/mL) were cultured in RPMI 1640 medium without red phenol (Gibco, USA) and were stimulated with synovial fluid obtained from RA or OA patients, PMA (40 nM, Solarbio), recombinant human IL-33 (the indicated concentrations, R&D Systems), or PBS for 4 h (or the indicated times) at 37 °C. Then, NET–DNA generated by neutrophils was digested with 1 U/mL micrococcal nuclease (Thermo Fisher Scientific) in the presence of 5 mM CaCl_2_ at 37 °C for 20 min, followed by the addition of 5 mM EDTA to stop nuclease activity. The supernatant was collected and centrifuged at 450 × *g* for 10 min to remove cell debris, followed by centrifugation at 12,000 × *g* for 10 min to remove cellular debris. Finally, NET–DNA concentration was determined with a Quant-iT PicoGreen dsDNA assay kit (Invitrogen) as described above. For NET preparation, no stimuli were used to coincubate neutrophils, and the remaining steps were consistent with the methods described above. The prepared NET–DNA complex was stored at −80 °C until further use.

In some assays, to inhibit NET formation, neutrophils were pretreated with DPI (10 μM, Selleck, USA), Cl-amidine (10 μM, MCE, USA), Sivelestat (20 μM, MCE), or the ST2 polyclonal antibody (0.2 mg/mL, Invitrogen).

### Live cell imaging experiments

Human neutrophils (2 ×10^5^ cells/well) were isolated and cultured in a 24-well plate with RPMI 1640 medium without red phenol (Gibco). Sytox green DNA dye (1:8000, Invitrogen) was added to each well to label dying cells (NETs). Live cell imaging was performed every 2 min using a Leica DMi8 live cell microscope with Leica X software (Leica, Germany).

### Immunofluorescence and scanning electron microscopy (SEM) analysis

Human neutrophils (2 ×10^5^ cells/well) were isolated and seeded on coverslips coated with poly-L-lysine in a 24-well plate and stimulated with PMA (40 nM, Solarbio), recombinant human IL-33 (100 ng/mL, R&D Systems), or PBS for 4 h at 37 °C. The cells were subsequently fixed with 4% paraformaldehyde, washed, permeabilized, and blocked. The tissues were stained with anti-histone H3 (citrulline R17) antibody in blocking buffer overnight at 4 °C, washed with PBS, and stained with Alexa Fluor 594-conjugated goat anti-rabbit IgG in blocking buffer at room temperature. DNA was stained with SYTOX Green DNA dye (1:8000, Invitrogen). Finally, the slides were mounted using Pro-Long Gold antifade reagent (Invitrogen) and observed via fluorescence microscopy (Olympus, Japan). Images were analyzed with FluoView software (Olympus). All the antibodies used were commercially validated, and the antibody usage information is provided in Supplementary Table 2.

For scanning electron microscopy analysis, the cells stimulated with PMA (40 nM, Solarbio) or IL-33 (100 ng/mL, R&D Systems) were fixed in electron microscope fixation liquid (Solarbio) for 2 h. SEM images and radiological scores of bone erosion were obtained by Wuhan Servicebio, Inc. (China).

### Cellular ROS detection

Neutrophils were stimulated with IL-33 (100 ng/mL, R&D Systems), PMA (40 nM, Solarbio) or PBS for 2 h. Cellular ROS levels in the neutrophils were then measured using the DCFDA/H2DCFDA Cellular ROS Assay Kit (Abcam, UK) following the manufacturer’s instructions. Finally, the ROS concentration was determined by measuring the fluorescence at 490/530 nm (excitation/emission) via a microplate reader.

### Coimmunoprecipitation (Co-IP)

Endogenous protein‒protein interactions in cells were examined using coimmunoprecipitation experiments with the Pierce™ Classic Magnetic IP/Co-IP Kit (Thermo Scientific) following the manufacturer’s protocol. Briefly, neutrophils stimulated with IL-33 (100 ng/mL, R&D Systems) or PBS for 1 h were lysed in cell lysis buffer containing 1 mM phenylmethylsulfonyl fluoride. The lysates were centrifuged, collected and incubated overnight at 4 °C with the anti-ST2 capture antibody, with continuous mixing throughout the incubation period. The following day, Pierce Protein A/G Magnetic Beads were added to the immunoprecipitated complexes and incubated for 1 h at room temperature. The magnetic beads were then collected, washed, and eluted. Following magnetic separation, the supernatant was collected, and the immunocomplexes present in the supernatant were analyzed via western blotting. All the antibodies used were commercially validated, and the antibody usage information is provided in Supplementary Table 2.

### RNA extraction and quantitative real-time polymerase chain reaction (qPCR)

Total RNA was isolated using the phenol/chloroform method with TRIzol Reagent (Ambion, USA). For mRNA quantification, cDNA was synthesized with the RevertAid First Strand cDNA Synthesis Kit (Thermo Fisher Scientific). Gene expression was subsequently measured using the TB Green Premix Ex Taq II Kit (Takara Biotechnology, China) on a QuantStudio DX Real-Time PCR Instrument with QuantStudio Dx Software (Applied Biosystems, USA). All operations were performed according to the reagent instructions. The sequences of the primers used in the study were designed via NCBI Primer-Blast (NCBI website) and are listed in Supplementary Table 3.

### Western blot analysis

Denatured protein samples were subjected to sodium dodecyl sulfate‒polyacrylamide gel electrophoresis (SDS‒PAGE, Beyotime, China) and then transferred to 0.2 μm PVDF membranes (Merck KGaA, Germany). Next, the PVDF membranes were blocked for at least 1 h in PBS/0.1% Tween-20 containing 5% skim milk at room temperature, followed by incubation with the primary antibody overnight at 4 °C. The next day, diluted HRP-conjugated secondary antibodies were added to the immunoblots and incubated at room temperature for at least 1 h. Finally, the proteins were visualized via enhanced chemiluminescence (ECL) reagents (Beyotime), and the immunoblot images were analyzed using Image Lab software (Bio-Rad Laboratories, Inc., USA). All the antibodies used were commercially validated, and the antibody usage information is provided in Supplementary Table 2.

### Flow cytometry

Peripheral blood cells or freshly isolated neutrophils from human participants were prepared for analysis. In some assays, neutrophils were stimulated with 100 ng/mL recombinant human IL-33 (R&D Systems) for 12 h. The cells were first incubated with PBS containing 1% bovine serum albumin (BSA, Solarbio) and 2% fetal bovine serum (FBS, Gibco) for 45 min to block Fc receptors. The samples were subsequently stained for the exclusion of dead cells using the LIVE/DEAD Fixable Dead Cell Stain Kit (Invitrogen). Next, the cell surfaces were stained for CD66 and ST2. Unstained and single-positive controls were used for gating and compensation. Flow cytometry was performed via a full-spectrum Cytek Northern Lights-CLC flow cytometer (CyteKios, China), and the results were analyzed via FlowJo software (version 10, FlowJo Inc.). All the antibodies used in the study are listed in Supplementary Table 2.

### Establishment and treatment of collagen antibody-induced arthritis (CAIA) mice

DBA/1 J mice were randomized into groups on the basis of age and weight. The mice were injected with 1.5 mg/mouse monoclonal antibody (mAb) cocktail against type II collagen (Chondrex, USA) via the tail vein on Day 0. On Day 3, LPS injection (50 µg) was performed to act synergistically with the mAb cocktail during arthritis development. Concurrently, mice were intraperitoneally injected with 1 μg of IL-33 (BioLegend, USA) per mouse for five consecutive days and/or administered intra-articular injections of 10 μL of spontaneously generated NETs or the TLR9 antagonist ODN 2088 (3 μM, InvivoGen, USA) into the knee joint. CAIA mice were then scored daily for clinical symptoms as previously described.^20^ Finally, the mice were anesthetized with 20% urethane (1 g/kg) and sacrificed by cervical dislocation on Day 9.

### Pathological and immunofluorescence staining and microcomputed tomography

After CAIA mice were sacrificed, the knee joints were promptly fixed in 4% phosphate-buffered formalin. The joints were subsequently decalcified in 10% EDTA for 30 days. The decalcified samples were then embedded in paraffin and sectioned. For histological analysis, the sections were stained with hematoxylin and eosin (H&E) to assess their general morphology, safranin-O-fast green-iron hematoxylin (Safranin O) to evaluate cartilage integrity, and toluidine blue staining to assess the proteoglycan content in the cartilage.

For IF staining, tyramide signal amplification (TSA) was utilized to observe the expression of specifically labeled proteins. Briefly, the paraffin sections were dewaxed, washed, subjected to antigen retrieval, and blocked. The primary antibody was added overnight at 4 °C, and the sections were then incubated with the corresponding HRP-conjugated secondary antibody at room temperature, followed by incubation with tyramide conjugates (Invitrogen). For double-label immunofluorescence, microwave treatment was employed to remove the antibody–TSA complex bound to the tissue sections, allowing for the introduction of the subsequent primary antibody and secondary antibody. Finally, DAPI (Roche, Germany) was used for cell nucleus counterstaining, and an anti-fluorescence quencher (Solarbio) was applied to seal the sections. All the antibodies used in the study are listed in Supplementary Table 2.

Microcomputed tomography (micro-CT) was utilized to reconstruct 3D images of the mouse joints. The knee joints of CAIA model mice were fixed in 4% phosphate-buffered formalin for 48 h. Samples were then sent to the Shanghai Experimental Animal Center (China) for subsequent processing, followed by micro-CT imaging and radiological scoring of bone erosion. The micro-CT images were scored for erosion according to the following criteria: 0 for normal, 1 for roughness, 2 for pitting, and 3 for full-thickness holes.

### Fibroblast-like synovial cell (FLS) isolation, culture and treatments

Primary RA FLSs were derived from fresh synovial tissue samples aseptically isolated from the joints of RA patients. Initially, synovial tissue samples were collected from the joints of RA patients undergoing routine synovectomy. The synovial tissue was then processed to remove fat and fibrous tissue, followed by cutting into fragments of approximately 1 cm^3^. These tissue fragments were enzymatically digested with 0.5 mg/mL collagenase I for 2 h at 37 °C in complete DMEM (Biosharp Co., Ltd., China). The resulting digested fragments were filtered through a 200-mesh sieve aseptically to isolate the digested cells. The isolated cells were subsequently cultured in complete DMEM containing 10% FBS (Gibco) for 24 h at 37 °C with 5% CO_2_, allowing adherence of the cells. After 24 h, fresh culture medium was added to remove nonadherent cells. Primary RA FLSs were further cultured and passaged using 0.25% trypsin/EDTA (Gibco) upon reaching approximately 80% confluence. Cells at passages 3–6 were utilized for the subsequent experiments.

In some experiments, RA FLSs were pretreated with specific inhibitors. The inhibitors used included the TLR2 antagonist TL2-C29 (20 μM, Invitrogen), the TLR4 antagonist CLI-095 (1 μM, InvivoGen), the TLR9 antagonist ODN TTAGGG (ODN A151, 3 μM, InvivoGen), the ERK inhibitor PD98059 (1 μM, Selleck), the p38 inhibitor SB203580 (10 μM, Selleck), the NF-κB inhibitor BAY11-7082 (10 μM, Selleck) and DNase I (0.1 mg/mL, Roche). Additionally, RA FLSs were also directly stimulated with spontaneously generated NETs at a concentration of 1 ng/μL, defined by NET-DNA concentration, for various durations, depending on the specific experimental design.

### RNA sequencing data analysis

The GSE150466 dataset was downloaded from the Gene Expression Omnibus (GEO) database (https://www.ncbi.nlm.nih.gov/geo/). Gene Ontology (GO), Kyoto Encyclopedia of Genes and Genomes (KEGG) and gene set enrichment analysis (GSEA) analyses were conducted using the clusterProfiler R package (version 4.8.3) in R (version R 4.3.1).

### Transwell migration assay

Transwell migration assays were conducted to assess neutrophil migration via a Transwell chamber (Millipore, USA). First, RA-FLSs were seeded in the bottom Transwell chamber and allowed to adhere for 24 h. Subsequently, 1 ng/μL spon-NETs were added to stimulate RA-FLSs for 24 h. Freshly isolated neutrophils were then added to the upper Transwell chamber. The upper Transwell chamber containing neutrophils was placed onto the bottom chambers and incubated for 5 h. Finally, the supernatant in the bottom Transwell chamber was collected, and neutrophil counts were measured using an auto hematology analyzer (BC-6800Plus, Mindray).

### Statistical analysis

Statistical analysis and plotting were performed using GraphPad Prism 9.3 (GraphPad software, Inc.) or R language (version R 4.3.1). In accordance with the normality of the data distribution, the data are presented as the means ± standard deviations (SDs)/standard errors of the means (SEMs) or medians (interquartile ranges), as indicated. For comparisons between two groups, Student’s *t* test or the Mann‒Whitney test was used, depending on the data distribution. Multiple t tests or multiple Mann‒Whitney tests with false discovery rate (FDR) correction (FDR < 0.01) were performed for multiple comparisons. Similarly, for comparisons among more than two groups, one-way analysis of variance (ANOVA) or the Kruskal‒Wallis test followed by Tukey’s or Dunn’s multiple comparisons test was conducted. Correlation analysis was conducted via Pearson correlation or Spearman correlation. Receiver operating characteristic (ROC) curves were calculated via the DeLong method. All the statistical tests were two-tailed, and a *p* value < 0.05 was considered significant (*p < 0.05, **p < 0.01, ***p < 0.001, ****p < 0.0001, ns not significant).

## Results

### NETs are increased in the RA synovial microenvironment and are associated with IL-33

**Fig. 1:**
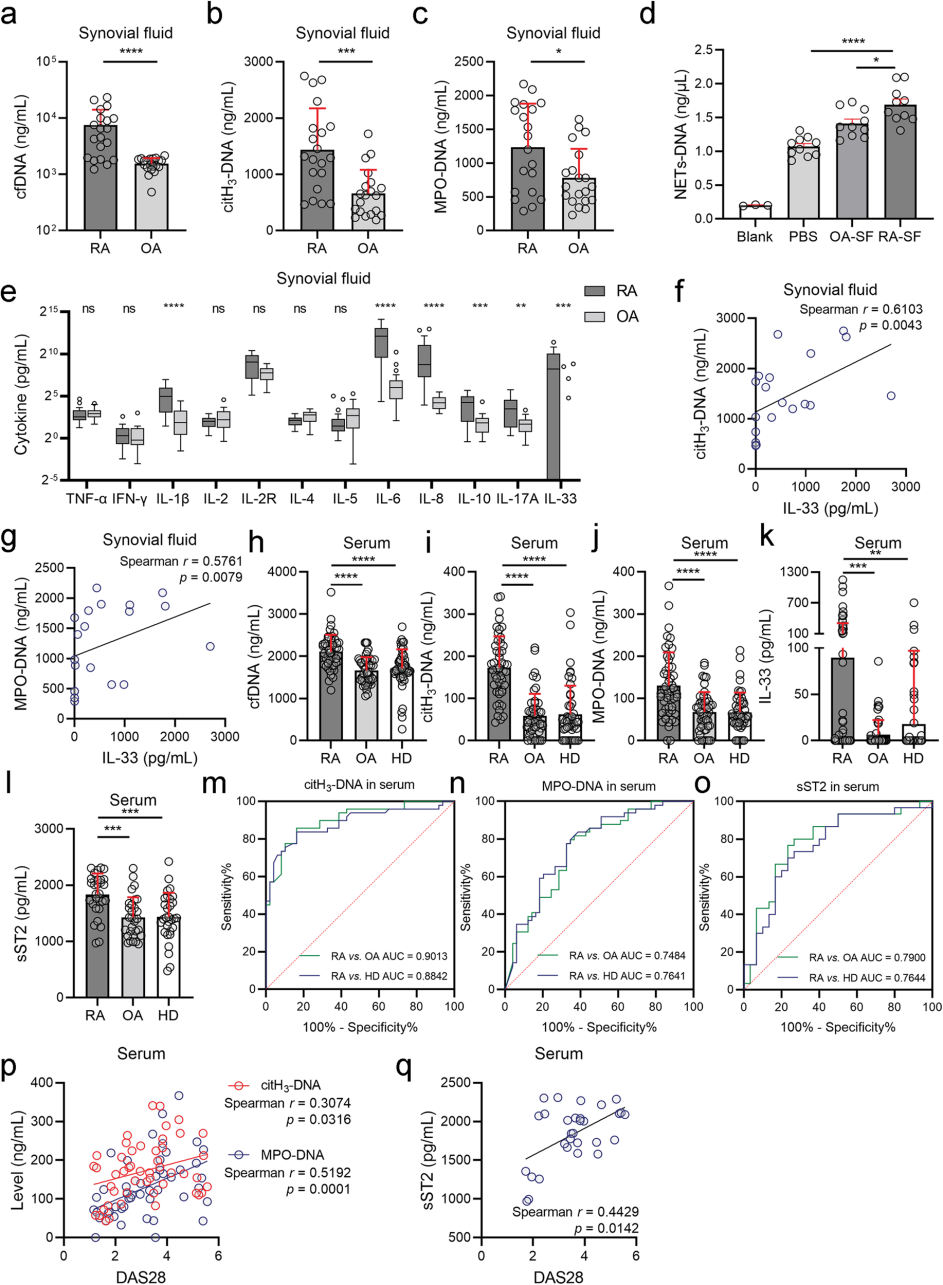
NETs are increased in the RA synovial microenvironment and associated with IL-33. **a**–**c** Cell-free DNA (cfDNA), citH_3_-DNA, and MPO-DNA levels in synovial fluid from RA (*n* = 20) and OA (*n* = 20) patients. **d** NET–DNA levels in HD neutrophils incubated with RA or OA synovial fluid for 4 h. **e** Levels of 12 cytokines in synovial fluid from RA (*n* = 20) and OA (*n* = 20) patients. **f**, **g** Correlations of citH_3_-DNA and MPO-DNA with IL-33 in RA synovial fluid (*n* = 20). **h**–**l** Peripheral serum levels of cfDNA (*n* = 49), citH_3_-DNA (*n* = 49), MPO-DNA (*n* = 49), IL-33 (*n* = 99 in RA patients, 62 in OA patients, 98 in HDs), and soluble ST2 (sST2) (*n* = 30) in peripheral serum from RA patients, OA patients, and HDs. **m**–**o** ROC curves of citH_3_-DNA (*n* = 49), MPO-DNA (*n* = 49), and sST2 (*n* = 30) distinguishing RAs from OAs or HDs. **p** Correlations of citH_3_-DNA and MPO-DNA with the disease activity score of 28 joints (DAS28) in the peripheral serum of individuals with RA (*n* = 49). **q** Correlation of sST2 with DAS28 in the peripheral serum of patients with RA (*n* = 30). All the data are presented as the means ± SDs; *p < 0.05, **p < 0.01, ***p < 0.001, ****p < 0.0001, ns not significant.

To investigate the potential role of NETs in the RA synovial microenvironment, we initially collected synovial fluid samples from patients with RA or OA. We then detected cell-free DNA (cfDNA), citH_3_-DNA and MPO-DNA using a modified ELISA method with MPO or citH_3_ serving as capture antibodies, along with the Quant-iT PicoGreen dsDNA detection kit. As expected, the levels of cell-free DNA (cfDNA), citH_3_-DNA and MPO-DNA in the synovial fluid of RA patients were significantly greater than those in the synovial fluid of OA patients (Fig. 1a–c). These findings suggest that certain components in RA synovial fluid potentially promote NET accumulation. To confirm this hypothesis, peripheral blood neutrophils isolated from healthy donors (HDs) were incubated with synovial fluid from RA or OA patients for 4 h, and NET-DNA levels were measured and normalized by subtracting the background levels in RA or OA synovial fluid. The results revealed that neutrophils incubated with RA synovial fluid generated more NETs than those incubated with OA synovial fluid did (Fig. 1d).

Given the role of cytokines in NET generation,^10^ we hypothesized that certain cytokines in RA synovial fluid might be responsible for NET accumulation. To verify this hypothesis, we examined the synovial fluid levels of 12 common cytokines and found that IL-1β, IL-6, IL-8, IL-10, IL-17A and IL-33 levels in RA synovial fluid were significantly greater than those in OA synovial fluid (Fig. 1e). Moreover, a correlation analysis between cytokines and NETs in RA synovial fluid was performed, revealing that IL-33 was significantly positively correlated with citH_3_-DNA and MPO-DNA (Fig. 1f, g). In contrast, other cytokines did not show such a correlation (Supplementary Table 4).

Next, we aimed to investigate whether the correlation between IL-33 and NETs was exclusive to the RA synovial microenvironment. Therefore, the levels of cfDNA, citH_3_-DNA, MPO-DNA, IL-33 and soluble stimulation 2 (ST2, the only known receptor of IL-33) were detected in the peripheral blood serum of patients with RA. Compared with OA patients and HDs, RA patients presented significantly higher serum cfDNA, citH_3_-DNA, MPO-DNA, IL-33 and soluble ST2 (sST2) levels (Fig. 1h–l). However, no correlation was detected between the levels of IL-33 and NETs, as well as sST2, in the serum of patients with RA (Supplementary Table 5). Additionally, we evaluated the potential clinical value of these serum indicators in RA. ROC analysis revealed that the serum citH_3_-DNA, MPO-DNA, and sST2 levels exhibited good discriminative ability between RA patients and either OA patients or HDs (Fig. 1m–o). Furthermore, these biomarkers were significantly correlated with disease severity in RA patients, as measured using the DAS28 scoring system (Fig. 1p, q and Supplementary Table 6). These findings suggest that serum citH_3_-DNA, MPO-DNA, and sST2 could serve as potential diagnostic markers for RA.

### IL-33 activates RA neutrophils to increase NET generation

**Fig. 2:**
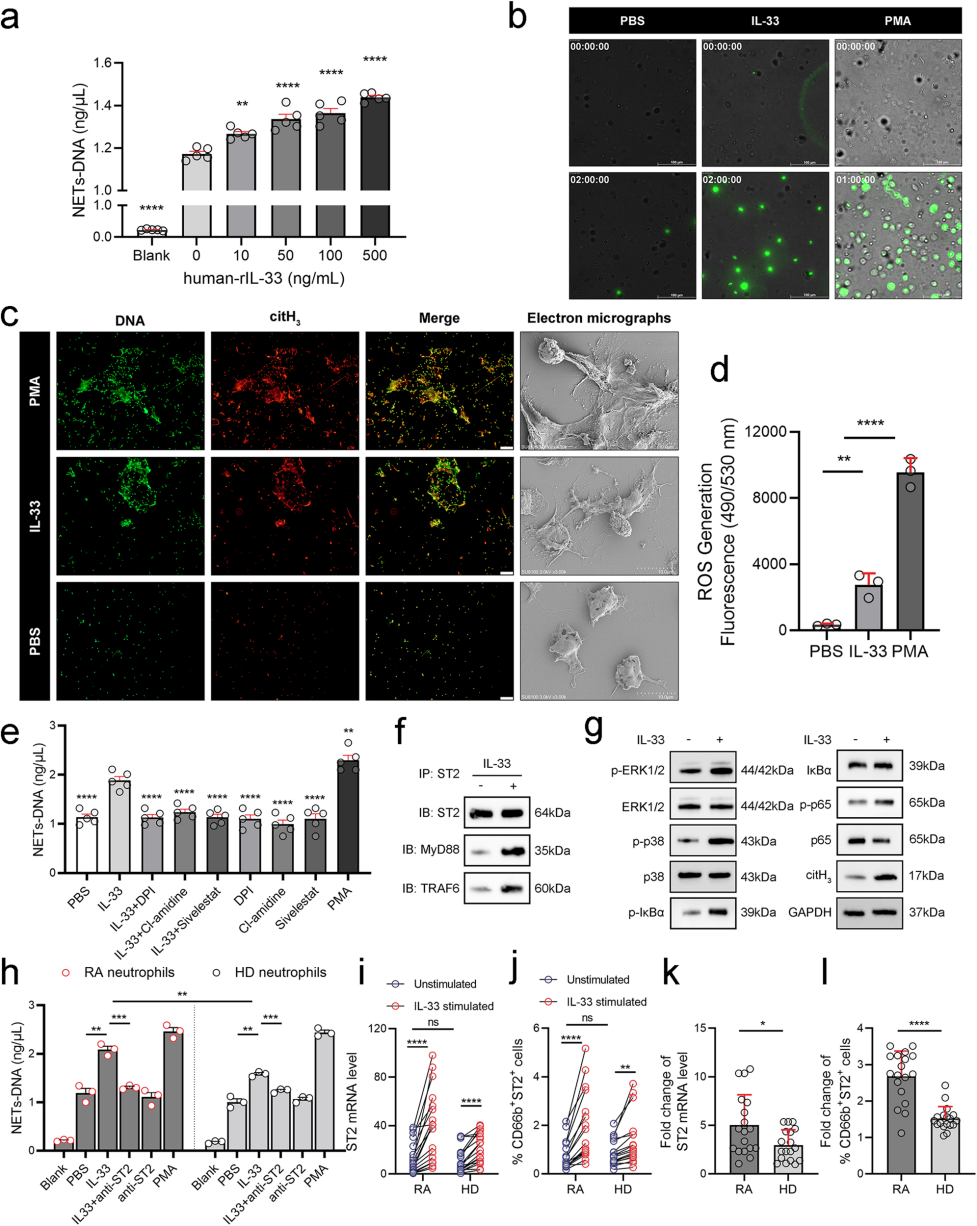
IL-33 activates RA neutrophils to increase NET generation. **a** NET–DNA levels produced from RA neutrophils incubated with various concentrations of IL-33 for 4 h. **b** Dynamic observation of NET formation in RA neutrophils induced with 100 ng/mL IL-33 using a live cell workstation. **c** Representative immunofluorescence staining and scanning electron microscopy images of NET formation induced by 100 ng/mL IL-33 in RA neutrophils. **d** Cellular ROS levels in neutrophils stimulated with 100 ng/mL IL-33, 40 nM PMA, or PBS for 2 h. **e** NET–DNA levels produced by RA neutrophils pretreated with 10 μM DPI, 10 μM Cl-amidine or 20 μM sivelestat for 1 h prior to stimulation with 100 ng/mL IL-33 or 40 nM PMA for 4 h. **f** Coimmunoprecipitation assay showing interactions among ST2, MyD88, and TRAF6 in RA neutrophils treated with 100 ng/mL IL-33 for 1 h. **g** Representative western blots showing ERK1/2, p38, IκBα and p65 phosphorylation levels in RA neutrophils incubated with 100 ng/mL IL-33 for 4 h. **h** NET–DNA levels induced from RA or HD neutrophils pretreated with 0.2 mg/mL ST2 polyclonal neutralizing antibody for 1 h and then stimulated with 100 ng/mL IL-33 or 40 nM PMA for 4 h. **i** ST2 mRNA levels in peripheral blood neutrophils from RA patients (*n* = 18) and HDs (*n* = 18) with or without incubation with 100 ng/mL IL-33 for 12 h. **j** Flow cytometric analysis of the proportion of CD66b+ST2+ cells among peripheral blood neutrophils from RA patients (*n* = 18) and HDs (*n* = 18) with or without incubation with 100 ng/mL IL-33 for 12 h. **k**, **l** Fold change in the ST2 mRNA level and the proportion of CD66b+ST2+ cells among RA (*n* = 18) or HD (*n* = 18) neutrophils incubated with 100 ng/mL IL-33 for 12 h. All the data are presented as the means ± SDs/SEMs. Asterisks represent significant differences compared with the reference group, which is without asterisks. *p < 0.05, **p < 0.01, ***p < 0.001, ****p < 0.0001, ns not significant.

Given the correlation between IL-33 and NETs in the RA synovial microenvironment, we wondered whether IL-33 contributes to NET formation in RA neutrophils. Therefore, neutrophils were isolated from the peripheral blood of RA patients, and an ex vivo experimental system in which different IL-33 concentrations were used to induce NET formation was established. As shown in Fig. 2a, NET formation gradually increased in an IL-33 concentration-dependent manner. Furthermore, dynamic observation of IL-33-induced NET formation was performed via a live cell workstation, and the results indicated that NET levels increased in a time-dependent manner with IL-33 induction (Fig. 2b and Supplementary Video 1–3). IL-33 could also stimulate NET formation in neutrophils from HDs, and this effect was both time- and concentration-dependent (Supplementary Fig. 1). Additionally, immunofluorescence staining and scanning electron microscopy revealed typical NET structures, indicating that, compared with the PBS control, IL-33 significantly increased NET generation (Fig. 2c).

It has been reported that ROS production is essential for the regulation of NET formation.^11,12^ Therefore, we analyzed ROS levels during the process of IL-33-triggered NET release using a DCFDA/H2DCFDA cellular ROS assay kit. The results showed that IL-33 induced ROS production in neutrophils (Fig. 2d). Next, we focused on the mechanism by which IL-33 facilitates NET formation. We pretreated RA neutrophils with classical NET inhibitors,^21^ including DPI (a NADPH oxidase inhibitor), Cl-amidine (a PAD4 inhibitor), or Sivelestat (an NE inhibitor), prior to stimulation with IL-33. All the inhibitors abrogated IL-33-induced NET formation, as confirmed by NET quantification (Fig. 2e), demonstrating that IL-33-induced NET formation is dependent on ROS, PAD4 and NE. Additionally, given that ST2 is the only known receptor for IL-33, we evaluated ST2 receptor activation in IL-33-treated neutrophils. Consistent with other studies,^22,23^ the Co-IP assay confirmed that the IL-33/ST2 interaction recruits the adaptor proteins MyD88 and TRAF6 (Fig. 2f), leading to the activation of the MAPK/ERK, p38 MAPK, and NF-κB signaling pathways in response to ROS production (Fig. 2g). Finally, neutrophils were pretreated with blocking antibodies against ST2 (anti-ST2) to block the IL-33 receptor ST2, resulting in significant inhibition of IL-33-induced NET formation, as expected (Fig. 2h).

The above results confirmed that IL-33 promoted NET generation in RA patients; next, we needed to further assess whether this effect was disease specific. Neutrophils were isolated from RA patients and HDs, and we observed that both RA and HD neutrophils were activated by IL-33. However, IL-33 could activate RA neutrophils to generate more NETs than HD neutrophils (Fig. 2h). We initially hypothesized that this difference might be due to varying numbers of ST2 receptors between RA and HD neutrophils. Unexpectedly, quantitative analysis using PCR and flow cytometry revealed that ST2 mRNA expression in neutrophils and the proportions of CD66b+ST2+ cells in peripheral blood did not significantly differ between RA patients and HDs (Fig. 2i, j). However, after RA or HD neutrophils were coincubated with IL-33 for 12 h, the ST2 mRNA levels and the proportions of CD66b+ST2+ cells among both types of neutrophils increased (Fig. 2i, j). Then, to account for interindividual differences in baseline levels, the fold increase was calculated by dividing the poststimulation value by the prestimulation value. The results revealed that both ST2 mRNA and ST2 protein levels were significantly greater in RA neutrophils than in HD neutrophils (Fig. 2k, l), indicating that RA neutrophils are more responsive to IL-33.

### IL-33 facilitates the progression of arthritis in collagen antibody-induced arthritis mice, accompanied by NET production

**Fig. 3:**
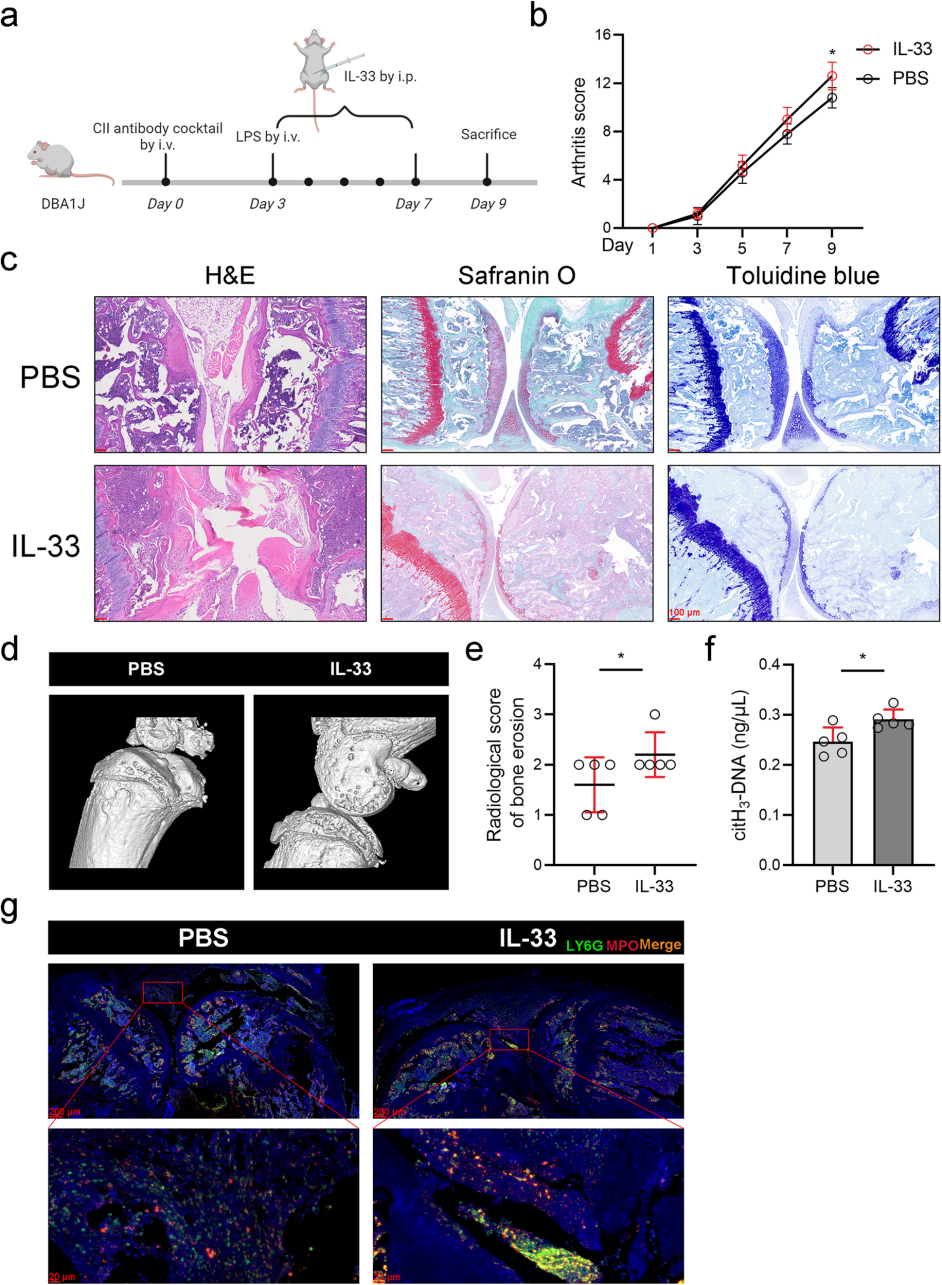
IL-33 facilitates the progression of arthritis in collagen antibody-induced arthritis mice, accompanied by NET production. **a** Flowchart of the animal experimental procedures created using BioRender.com. DBA/1J mice received 1.5 mg/mouse type II collagen mAbs via the tail vein on Day 0. On Day 3, 50 µg of LPS was injected via the tail vein to synergize with the mAbs for arthritis development. The mice were also administered 1 µg of IL-33 intraperitoneally for five consecutive days. **b** Inflammatory scores of CAIA mice treated with IL-33 (*n* = 5) or the solvent control (*n* = 5). **c** Representative H&E, safranin O, and toluidine blue staining of joint tissue from IL-33-treated CAIA mice. **d**, **e** Representative micro-CT images and radiological scores of bone erosion in IL-33-treated CAIA mice. **f** Peripheral serum levels of citH_3_-DNA in CAIA mice. **g** Representative immunofluorescence image of joint tissue from IL-33-treated CAIA mice. All the data are presented as the means ± SDs, *p < 0.05.

To further validate the role of IL-33 in RA, we performed validation experiments using a collagen antibody-induced arthritis (CAIA) mouse model. As shown in the animal experimental flowchart displayed in Fig. 3a, CAIA mice were subjected to continuous intraperitoneal injection of 1 μg of IL-33 from Day 3 to Day 7, whereas the IL-33 solvent (PBS) served as a control. CAIA model mice were sacrificed on Day 9, when arthritis had reached its maximum severity.^20^ Joint inflammation in CAIA model mice was scored every 2 days starting on Day 1, and we noted that the inflammation scores were significantly increased in CAIA model mice treated with IL-33 (Fig. 3b). Moreover, H&E staining, Safranin O staining and toluidine blue staining indicated that inflammatory cell infiltration, synovial hyperplasia and cartilage destruction were more severe in the IL-33-treated mice than in the PBS control mice (Fig. 3c). Additionally, representative 3D reconstructed images of the knee joints of CAIA mice obtained via microcomputed tomography (micro-CT) analysis revealed radiographic features. Radiological scores of bone erosion revealed that IL-33 facilitates bone erosion in CAIA mice (Fig. 3d, e). Finally, a modified ELISA with a citH_3_ capture antibody was used to measure citH_3_-DNA levels in the peripheral blood serum of CAIA mice. Immunofluorescence staining of mouse joint tissues was also performed to measure NET levels in the joints. The results demonstrated that NET levels were increased in IL-33-treated CAIA mice, regardless of whether NETs were present in the peripheral blood or the joint cavity microenvironment (Fig. 3f, g).

### NETs enhance IL-33 production and neutrophil recruitment by activating fibroblast-like synovial cells

**Fig. 4:**
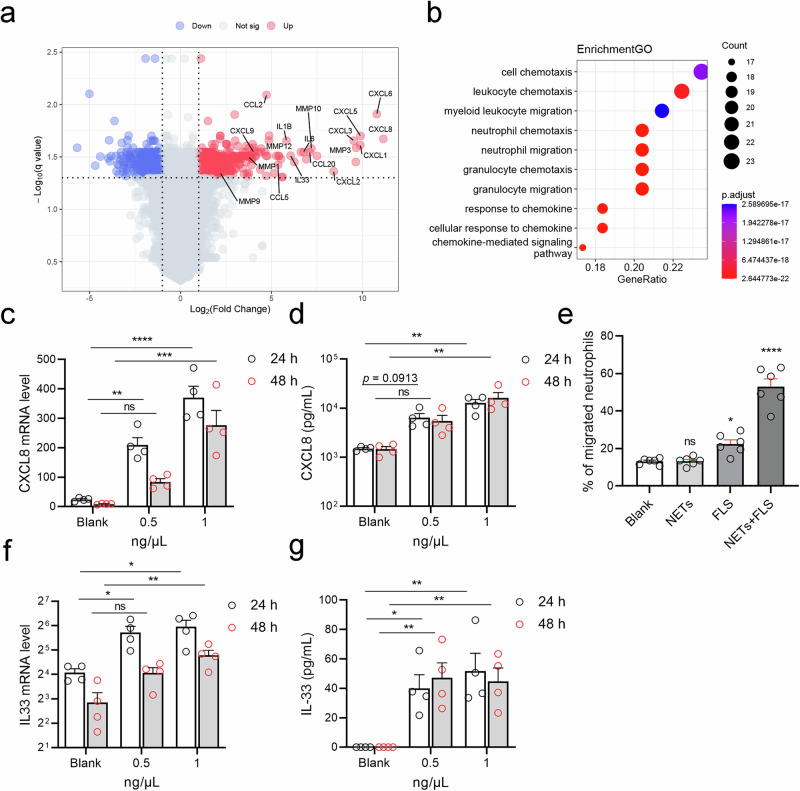
NETs enhance IL-33 production and neutrophil recruitment through fibroblast-like synovial cells. **a** Volcano plot of genes differentially expressed between NET-treated FLSs and control FLSs. **b** GO annotation analysis of DEGs between NET-treated FLSs and control FLSs. **c**, **d** CXCL8 mRNA and protein levels in FLSs (*n* = 4) incubated with 0.5/1 ng/μg spontaneously generated NETs (spon-NETs) for 24–48 h. **e** Neutrophil migration was measured using the Transwell migration assay (*n* = 6). **f**, **g** IL-33 mRNA and protein levels in FLSs (*n* = 4) incubated with 0.5/1 ng/μg spon-NETs for 24/48 h. All data are presented as the means ± SEMs. Asterisks represent significant differences compared with the reference group, without asterisks. *p < 0.05, **p < 0.01, ***p < 0.001, ****p < 0.0001, ns = not significant.

We confirmed that increased IL-33 promoted NET formation in the RA synovial microenvironment; however, the function of NETs in joints affected by RA remains to be further explored. Considering the pathophysiological role of fibroblast-like synovial cells (FLSs) in RA,^24^ to analyze the effects of NETs on RA-FLSs, we downloaded the GSE150466 dataset from the GEO database. This dataset includes bulk RNA sequencing data from FLSs treated with NETs for 48 h. Subsequently, the raw data were processed and plotted via a volcano plot based on the fold changes and corrected *p* values. We observed that the gene expression of many inflammatory cytokines, chemokines and metal matrix proteins was upregulated in NET-stimulated FLSs. Notably, this included IL-33 and the neutrophil chemokine CXC motif chemokine ligand 8 (CXCL8, also known as IL-8) (Fig. 4a). GO (Fig. 4b), KEGG, and GSEA (Supplementary Fig. 2a, b) enrichment analyses revealed that the differentially expressed unigenes were significantly enriched in granulocyte chemotaxis- and cytokine activity-associated subcategories. Next, we isolated and cultured FLSs from the synovial tissues of RA patients who underwent synovectomies. Then, spontaneously generated NETs (spon-NETs) were added to the culture medium of FLSs for 24 or 48 h, after which the cells and supernatant were collected. The results of quantitative PCR revealed that CXCL8 mRNA expression levels in FLSs increased with increasing concentrations of spon-NETs, peaking at 24 h (Fig. 4c). CXCL8 protein expression in the supernatant also increased with increasing NET concentration and duration of NET coculture (Fig. 4d). These results suggest that NET-activated FLSs are likely to recruit neutrophils. Therefore, we examined the ability of NET-activated FLSs to recruit neutrophils via a Transwell migration assay. As shown in Fig. 4e, cocultures of spon-NETs and RA-FLSs significantly promoted neutrophil migration compared with the other control groups. Like those of CXCL8, the IL-33 mRNA and protein expression levels in RFA-FLSs also significantly increased after spon-NET stimulation (Fig. 4f, g). These results suggest that a positive feedback loop may exist in vivo, whereby IL-33 promotes NET formation in the RA synovial microenvironment. This, in turn, stimulates RA-FLSs to produce IL-33 and recruit neutrophils.

### NETs activate fibroblast-like synovial cells via Toll-like receptor 9

**Fig. 5:**
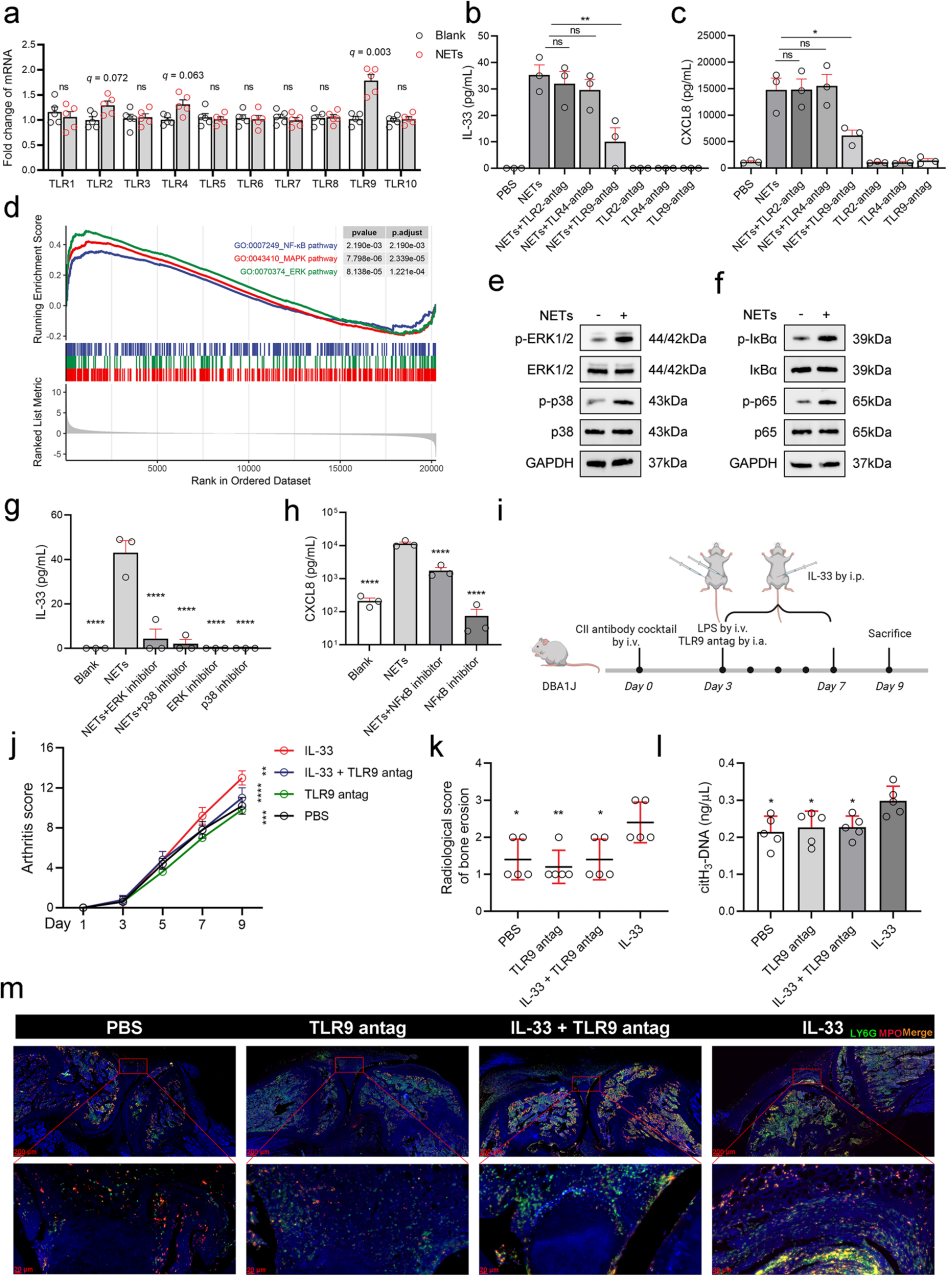
NETs activate fibroblast-like synovial cells via Toll-like receptor 9. **a** TLR1 to TLR10 mRNA levels in RA FLSs (*n* = 5) treated with 1 ng/μg spon-NETs. **b**, **c** Levels of IL-33 and CXCL8 secreted from RA FLSs (*n* = 3) preincubated with 20 μM TLR2 antagonist TL2-C29, 1 μM TLR4 antagonist CLI-095 and 3 μM TLR9 antagonist ODN TTAGGG (ODN A151) for 1 h prior to the addition of 1 ng/μL spon-NETs for 24 h. **d** GSEA of the NF-κB and MAPK (ERK1/2) signaling pathways. **e**, **f** Representative western blots showing the levels of phosphorylated ERK1/2, p38, IκBα and p65 in RA neutrophils incubated with 1 ng/μL spon-NETs for 24 h. **g**, **h** Levels of IL-33/CXCL8 secreted from RA FLSs (*n* = 3) preincubated with the ERK inhibitor PD98059 (1 μM) or the p38 inhibitor SB203580 (10 μM) or the NF-κB inhibitor BAY11-7082 (10 μM) for 1 h prior to the addition of 1 ng/μL spon-NETs for 24 h. **i** Flowchart of the animal experimental procedures created using BioRender.com. DBA/1J mice received 1.5 mg/mouse type II collagen mAbs via the tail vein on Day 0. On Day 3, 50 µg of LPS was injected via the tail vein to synergize with the mAbs for arthritis development, and intra-articular injections of 3 μM TLR9 antagonist ODN 2088 were administered into the knee joint. The mice were also administered 1 µg of IL-33 intraperitoneally for five consecutive days. **j** Inflammatory scores of IL-33-induced CAIA mice treated with a TLR9 antagonist (*n* = 5) and control mice (*n* = 5). **k** Radiological scores of bone erosion in IL-33-induced CAIA mice treated with a TLR9 antagonist and controls. **l** citH_3_-DNA levels in the peripheral serum of CAIA model mice treated with a TLR9 antagonist and control mice. **m** Representative immunofluorescence staining of joint tissue from IL-33-induced CAIA mice treated with a TLR9 antagonist. All the data are presented as the means ± SEMs/SD. Asterisks represent significant differences compared with the reference group, without asterisks. *p < 0.05, **p < 0.01, ***p < 0.001, ****p < 0.0001.

The mechanisms by which RA FLSs prime the release of the cytokines IL-33 and CXCL8 through NETs were further investigated. Given that Toll-like receptor (TLR) signaling is a major pathway for initiating and maintaining proinflammatory cytokine production,^25^ we examined the changes in the mRNA expression of TLR1 to TLR10 in RA FLSs treated with spon-NETs. The results revealed that the changes in TLR2 (q value = 0.072) and TLR4 mRNA expression (q value = 0.063) approached statistical significance, whereas TLR9 (q value = 0.003) mRNA levels increased in FLSs treated with spon-NETs (Fig. 5a). To avoid missing potential receptors, TLR2, TLR4 and TLR9 were included in subsequent experiments. RA-FLSs were preincubated with the TLR2 antagonist TL2-C29, the TLR4 antagonist CLI-095 and the TLR9 antagonist ODN TTAGGG (ODN A151) for 1 h prior to the addition of spon-NETs and then further incubated for 24 h. IL-33 and CXCL8 levels were detected in the cell supernatant. We found that antagonizing TLR9 in FLSs significantly inhibited IL-33 and CXCL8 expression, whereas antagonizing TLR2 or TLR4 did not (Fig. 5b, c). These findings indicated that NETs promoted cytokine production in FLSs via the TLR9 signaling pathway. The increased IL-33 and CXCL8 production in spon-NET-activated FLSs prompted us to evaluate MAPK (ERK1/2)^26^ pathway and the NF-κB signaling pathway activation.^27^ GSEA suggested that the MAPK (ERK1/2) and NF-κB signaling pathways were activated in NET-activated FLSs (Fig. 5d). Moreover, the western blot results confirmed the above analysis, showing increased phosphorylation of ERK1/2, p38, IκBα and p65 following the administration of spon-NETs (Fig. 5e, f). In addition, the ERK inhibitor PD98059 and the p38 inhibitor SB203580 significantly suppressed IL-33 expression in NET-activated FLSs (Fig. 5g), whereas the NF-κB inhibitor BAY11-7082 inhibited CXCL8 secretion (Fig. 5h).

Finally, we evaluated the efficacy of the mouse TLR9 antagonist ODN2088 in mitigating IL-33-induced arthritis in CAIA mice. CAIA model mice were injected with the TLR9 antagonist via intra-articular injection before the first intraperitoneal injection of IL-33 on Day 3 (Fig. 5i). The inflammation score was first calculated, and we found that the TLR9 antagonist attenuated IL-33-induced arthritis in CAIA mice (Fig. 5j). H&E staining, Safranin O staining, and toluidine blue staining revealed that the TLR9 antagonist inhibited IL-33-induced inflammatory cell infiltration, synovial hyperplasia, and cartilage destruction in the joints (Supplementary Fig. 3a). Representative micro-CT images of knee joints from CAIA mice (Supplementary Fig. 3b) and radiological scores of bone erosion (Fig. 5k) also revealed that the TLR9 antagonist effectively attenuated IL-33-induced articular bone damage in CAIA mice. Furthermore, modified ELISA and immunofluorescence staining of mouse joint tissues confirmed that NET levels were decreased in the peripheral blood and in the joint cavity microenvironment of IL-33-treated CAIA mice challenged with a TLR9 antagonist (Fig. 5l, m). Finally, PCR analysis of the synovial tissue of IL-33-treated CAIA mice revealed that the TLR9 antagonist decreased *Tlr9*, *Il33*, *Il6*, and *Tnf* mRNA expression (Supplementary Fig. 3c).

### NET-activated fibroblast-like synovial cells contribute to increased NET formation in vitro and in vivo

**Fig. 6:**
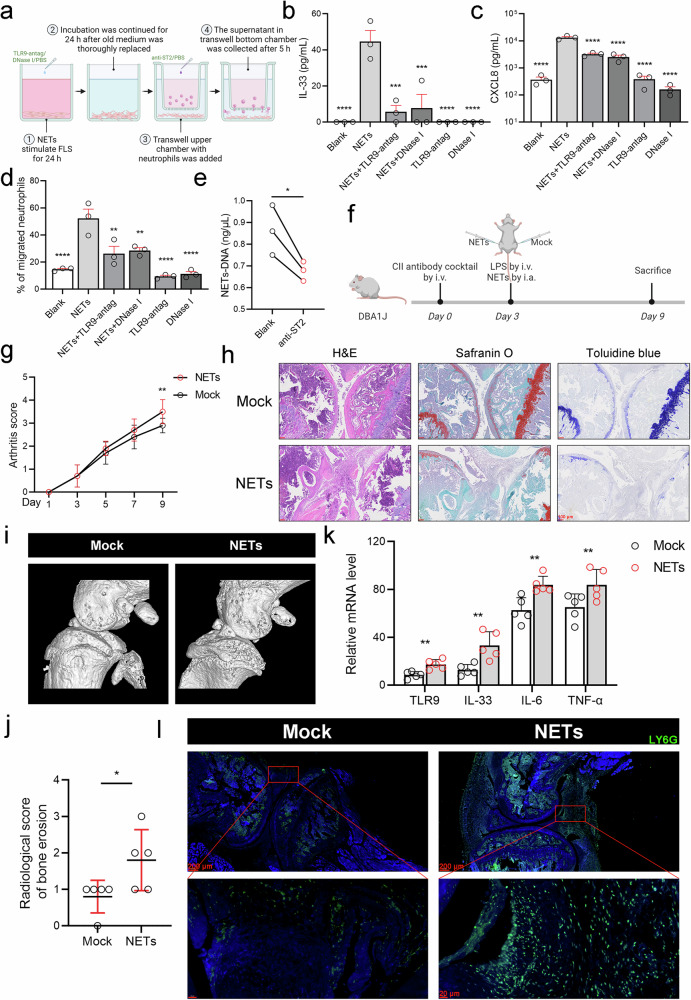
NET-activated fibroblast-like synovial cells contribute to increased NET formation in vitro and in vivo. **a** Schematic of the modified migration Transwell assay, which was created with BioRender.com. **b**, **c** Levels of IL-33 and CXCL8 secreted from RA FLSs (*n* = 3) preincubated with 3 μM TLR9 antagonist ODN TTAGGG (ODN A151) or 0.1 mg/mL DNase I in the modified Transwell assay. **d** Neutrophil migration was measured using the modified Transwell migration assay (*n* = 3). **e** NET–DNA levels in the Transwell chamber (*n* = 3). **f** Flowchart of the animal experimental procedures created with BioRender.com. DBA/1 J mice received 1.5 mg/mouse type II collagen mAbs via the tail vein on Day 0. On Day 3, 50 µg of LPS was injected via the tail vein to synergize with the mAbs for arthritis development, and 10 μL of spon-NETs was intra-articularly injected into the right knee joint, and the solvent (RPMI 1640 medium) control was administered into the left knee joint. **g** Inflammatory scores of individual joints in CAIA mice treated with 1 ng/μg spon-NETs (*n* = 10) or the control (*n* = 10). **h** Representative H&E, safranin O, and toluidine blue staining of joint tissue from spon-NETs-treated CAIA mice (*n* = 5). **i**, **j** Representative micro-CT images and radiological scores of bone erosion in spon-NET-treated CAIA mice (*n* = 5). **k** *Tlr9*, *Il33*, *Il6*, and *Tnf* mRNA levels in spon-NET-treated CAIA mice (*n* = 5). **l** Representative immunofluorescence image of joint tissue from spon-NET-treated CAIA mice (*n* = 5). All the data are presented as the means ± SEMs/SD. Asterisks represent significant differences compared with the reference group, without asterisks. *p < 0.05, **p < 0.01, ***p < 0.001, ****p < 0.0001.

We designed a modified Transwell migration assay to evaluate the potential positive feedback loop between NETs and RA-FLSs in vitro. A schematic representation is shown in Fig. 6a. RA-FLSs were cocultured with spon-NETs in the Transwell bottom chamber for 24 h, followed by removal of the old medium. Then, fresh medium was added, and the mixture was incubated for the next 24 h. Finally, the upper Transwell chamber with neutrophils was moved to the bottom chambers, and the supernatant in the bottom Transwell chamber was collected to evaluate cytokine secretion and neutrophil migration. We noted that spon-NET-activated RA FLSs promoted IL-33 and CXCL8 secretion and neutrophil recruitment. However, when RA FLSs were preincubated with a TLR9 antagonist or DNase I prior to the beginning of the experiment, cytokine secretion and neutrophil recruitment were significantly suppressed (Fig. 6b–d). Moreover, NET–DNA levels decreased in the Transwell bottom chamber if the ST2 receptor of neutrophils in the Transwell upper chamber was blocked in advance by ST2-blocking antibodies (Fig. 6e).

To determine whether NETs have pathogenic effects on the RA synovial microenvironment, CAIA mice were challenged on Day 3 by intra-articular injection of 10 μL of spon-NETs into the right knee joint and solvent (RPMI 1640 medium) control into the left knee joint (Fig. 6f). The inflammation score of a single joint was calculated, and the results demonstrated that the scores of the joints injected with NETs were greater than those of the joints injected with the control solvent (Fig. 6g). H&E staining, Safranin O staining, and toluidine blue staining also confirmed that NETs exacerbated inflammatory cell infiltration, synovial hyperplasia, and cartilage destruction in the joints (Fig. 6h). Representative micro-CT images of the knee joints of CAIA mice and radiological scores of bone erosion indicated that NETs facilitate articular bone damage in CAIA mice (Fig. 6i, j). The results of the PCR analysis of synovial tissue from NET-treated CAIA mice revealed that NETs increased *Tlr9*, *Il33*, *Il6*, and *Tnf* mRNA levels (Fig. 6k). Moreover, immunofluorescence staining of mouse joint tissues revealed that many neutrophils infiltrated the knee joint cavity previously injected with NETs (Fig. 6l).

## Discussion

Increasing evidence shows that neutrophils play a pivotal role in RA by breaking immune tolerance through NET formation.^8,28^ Although various stimuli are reported to promote NET formation, the precise mechanisms underlying NET induction in RA-inflamed joints remain unclear. In this study, we first evaluated and confirmed that compared with OA synovial fluid, neutrophils incubated with RA synovial fluid generated a greater number of NETs. Given the unique characteristics of RA, our investigation focused on cytokines present in the synovial fluid of RA patients. We discovered that both IL-33 and NET levels were elevated in RA synovial fluid and exhibited a robust correlation, indicating that increased IL-33 promoted NET formation in the RA synovium. Subsequent experiments further confirmed that IL-33-mediated NET formation occurred in a time- and concentration-dependent manner.

IL-33, a cytokine belonging to the IL-1 superfamily, is widely expressed throughout the body and plays crucial roles in tissue development, homeostasis, and repair.^29^ It has been reported to play dual roles as an anti-inflammatory and pro-inflammatory cytokine in immunity through its specific receptor ST2.^23^ Alternative splicing of ST2 generates soluble ST2, which competes with membrane-bound ST2 to negatively regulate IL-33.^29^ In our study, we confirmed previous reports of elevated IL-33 levels in RA patients.^30–32^ Although IL-33 is not detected in all RA patients possibly owing to its short half-life^33^ and rapid degradation mechanisms,^34^ the elevated serum sST2 levels in RA patients suggest that the negative regulation of IL-33 has been activated to counteract its increased levels. Moreover, ROC curve analysis revealed that serum citH_3_-DNA and sST2 may serve as potential diagnostic biomarkers for RA. The observed correlations between these indicators and DAS28 scores further highlight their potential clinical relevance. Additionally, we also observed that RA neutrophils were more responsive to IL-33 stimulation, leading to increased NET formation. Other studies have reported elevated ST2 receptor levels in RA neutrophils compared with those in HDs.^33^ Unexpectedly, our research did not observe this difference, which may be attributed to variations in ethnicity, region, and treatment regimens among the study participants. Nonetheless, we found that RA neutrophils presented greater ST2 expression in response to IL-33 stimulation than did neutrophils from HDs. These findings suggest that the heterogeneous genetic background between RA patients and healthy controls may affect ST2 expression and/or that the epigenetic features of RA neutrophils are altered during RA progression.

The role of IL-33 in RA remains somewhat controversial. IL-33 can act as either an anti-inflammatory factor or a proinflammatory factor, which leads to opposite results for IL-33 in RA.^35^ For example, IL-33 induces neutrophil migration by activating macrophages, and the suppression of ST2 expression in neutrophils by an anti-TNF-α antibody (infliximab) may represent a critical mechanism underlying the anti-inflammatory effects of TNF-α therapy in RA.^36^ Additionally, it has been suggested that IL-33 exacerbates antigen-induced arthritis by activating mast cells in collagen-induced arthritis (CIA) mice and that blocking IL-33 alleviates joint inflammation in these mice.^37–39^ In contrast, the results of one study revealed that repeated treatment with IL-33 in CIA mice, both at early and late stages of the disease, attenuated inflammation rather than exacerbated it.^40^ This protective effect may be attributed to the expansion of type 2 innate lymphoid cells and regulatory T cells, as well as a shift toward a Th2 cell response.^40^ The in vivo results of our study indicated that the intraperitoneal administration of IL-33 facilitates the infiltration of inflammatory cells, synovial hyperplasia, cartilage destruction, and bone erosion in CAIA mice. Additionally, NETs were observed in the joints. These results support the notion that IL-33 promotes NET formation in vivo and plays a pathogenic role in RA.

Synovial inflammation and hyperplasia with pannus formation, as well as bone and cartilage destruction, are the fundamental pathological features of RA.^1,4^ Fibroblast-like synoviocytes (FLSs) are a key component of the synovial pannus, displaying a uniquely aggressive phenotype and actively contributing to the pathological progression of RA.^41^ In the RA synovium, FLSs can function as antigen-presenting cells, internalize NET products, upregulate MHC class II molecules, and present NET-derived peptides to CD4 + T cells,^42^ establishing an association with the adaptive immune response in RA. Given the accumulation of NETs in inflamed joints in RA patients and the importance of FLSs as important components in the RA synovium, this study further investigated the interaction between FLSs and NETs. The results demonstrated that NET-activated FLSs secreted significant amounts of IL-33 and CXCL8, which subsequently recruited and activated neutrophils to produce more NETs. Mechanistically, we confirmed that NETs activated FLSs via TLR9, an intracellular innate immune receptor that recognizes pathogens and self-DNA in immune complexes.^43^ This activation subsequently triggers the MAPK (ERK1/2) and NF-κB signaling pathways, upregulating IL-33 and CXCL8 secretion. Furthermore, a TLR9 antagonist reduced IL-33-induced arthritis in CAIA mice in vivo. Finally, an in vitro modified Transwell migration assay suggested a positive feedback loop in which NETs regulate their levels through FLSs in the RA synovium. Injecting NETs into the knee joints of CAIA mice accelerated arthritis progression and increased neutrophil infiltration. Numerous studies have reported elevated IL-33 and NET levels in individuals with RA.^22,44^ Additionally, NETs activate FLSs,^45^ and IL-33 produced by FLS activate neutrophils through both direct and indirect mechanisms.^46,47^ However, no studies have linked these three factors in the context of RA to date. Our study elucidates the interrelationship among IL-33, NETs, and FLSs in the RA synovial microenvironment, with a particular emphasis on the amplifying role of FLSs and IL-33 in NET formation. Overall, these data further support the notion that the interaction between FLSs and NETs in vivo has pathogenic consequences, indicating the existence of an amplifying mechanism for NETs in the RA synovium. This mechanism leads to the release of many modified proteins and PADs. These PADs can modify proteins in the extracellular space, thus creating additional epitopes for autoantibody ACPA generation. In addition, research has shown that IL-33 is processed into mature bioactive forms by neutrophil elastase and cathepsin G,^48^ both of which are abundant in neutrophils. Furthermore, NETs released from systemic lupus erythematosus (SLE) neutrophils contain bioactive IL-33, and these NETs decorated with bioactive IL-33 stimulate robust IFN-α synthesis.^49^ The research mentioned above, along with our results, demonstrates a close association of IL-33 with NETs in autoimmune diseases.

Despite substantial supporting evidence for the involvement of neutrophils in various stages of RA pathogenesis and their natural history,^8^ therapeutic strategies that specifically target neutrophil dysregulation may not be the optimal choice. This is in part explained by the profound immunosuppressive effects of targeting neutrophils in terms of increasing infection susceptibility.^28^ Inhibiting NET formation or the process of NET formation and amplification is a particularly attractive strategy in RA because these approaches do not directly affect neutrophil numbers or crucial neutrophil functions. Furthermore, some evidence suggests that impaired NET formation does not lead to significant general immunosuppression.^12^

**Fig. 7:**
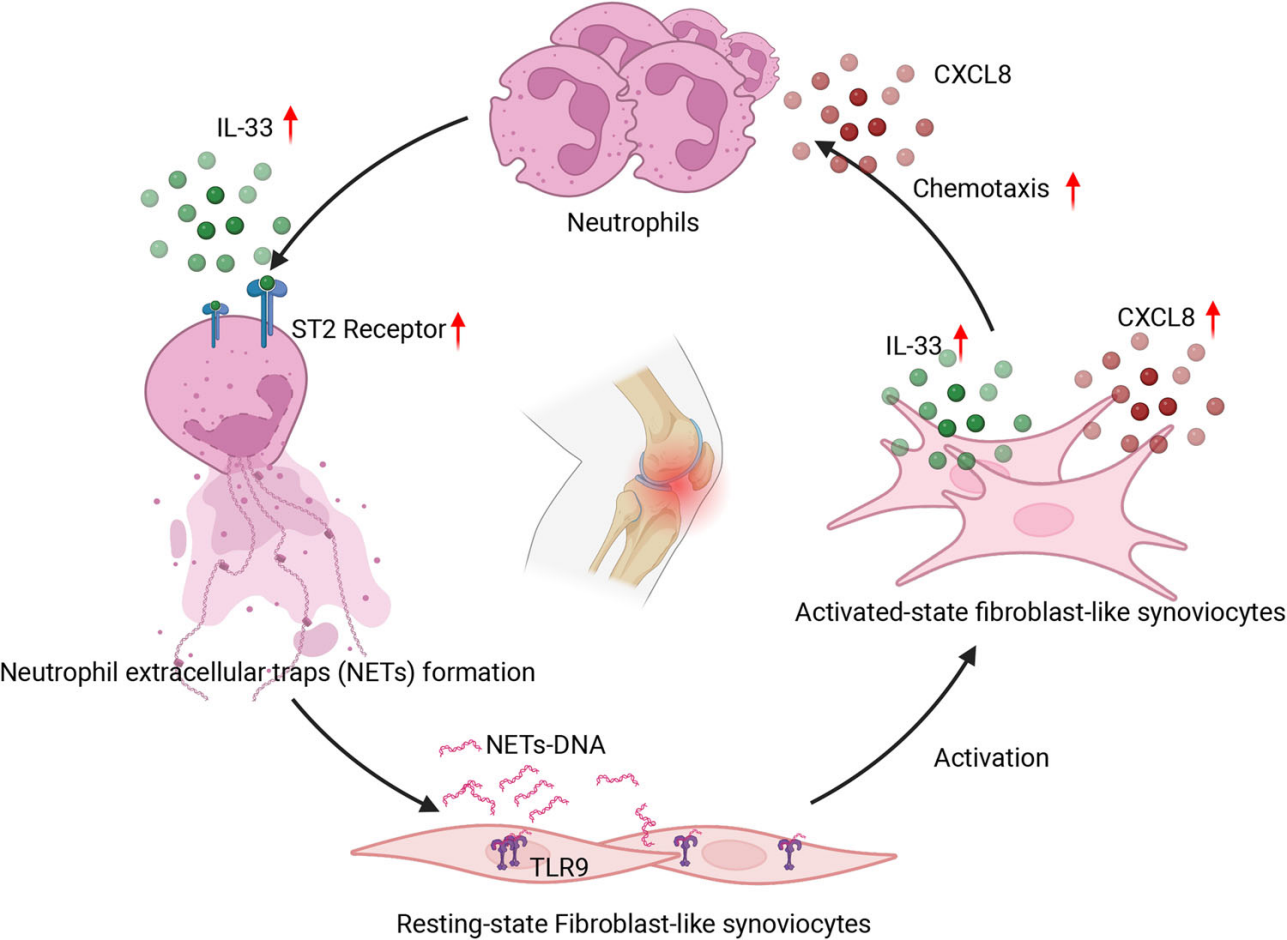
Schematic diagram of positive feedback loop in synovial inflammation and NET amplification in RA. Schematic Diagram shows how IL-33 drives a positive feedback loop, increasing NET formation and synovial inflammation in RA. Created using BioRender.com.

In summary, this study provides both in vitro and in vivo evidence that increased IL-33 promotes NET formation in the RA synovial microenvironment. Furthermore, NETs stimulate FLSs, which subsequently recruit neutrophils to generate more NETs through the release of IL-33 and CXCL8 (Fig. 7). These findings reveal a novel mechanism for the involvement of IL-33 in the pathogenesis of RA and the amplification mechanism of NETs in the RA synovium. Thus, targeting IL-33 or the process of NET formation and amplification represent a potential therapeutic strategy for RA treatment.

## Supplementary information

Supplementary Information

Supplementary Video 1: live cell workstation-IL-33

Supplementary Video 2: live cell workstation-PBS

Supplementary Video 3: live cell workstation-PMA

## Data Availability

The publicly available data utilized in this paper can be accessed through the Gene Expression Omnibus (GEO) database under the accession number GSE150466. The GEO is a global and publicly accessible online repository for gene expression data.
