# Supplementary material for: IL33-induced neutrophil extracellular traps (NETs) mediate a positive feedback loop for synovial inflammation and NET amplification in rheumatoid arthritis
Source: Exp Mol Med. 2024 Dec 2;56(12):2602–16. doi: 10.1038/s12276-024-01351-7 (PMC11671579; doi:10.1038/s12276-024-01351-7)
Supplement: Supplementary file 1 — Supplementary Information [file 12276_2024_1351_MOESM1_ESM.pdf]

Supplementary Fig. 1

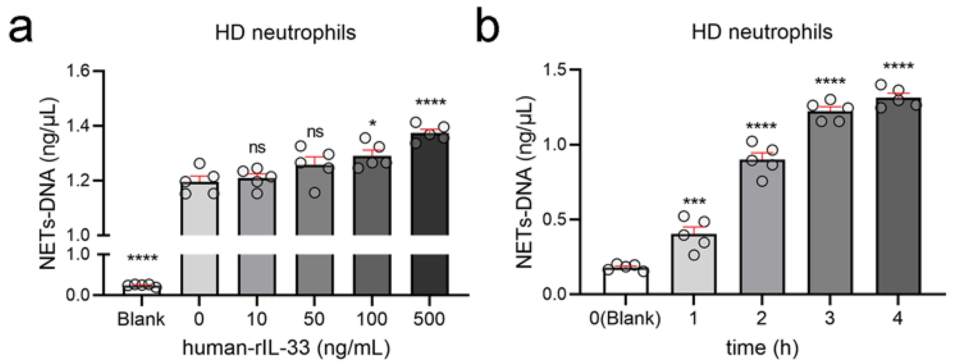

Supplementary Fig. 1. IL-33 activates HD neutrophils to generate NETs. (**a**) Levels of NETs-DNA induced from HD neutrophils incubated with varying concentrations of IL-33 for 4 h. (**b**) Levels of NETs-DNA induced from HD neutrophils incubated with 100 ng/mL IL-33 for varying durations.

Supplementary Fig. 2

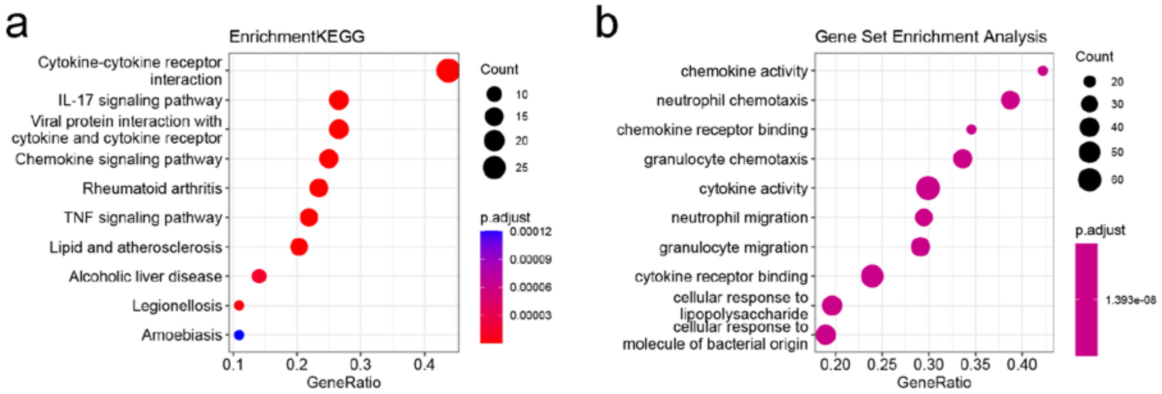

Supplementary Fig. 2. NETs enhance chemokine production and neutrophil recruitment through FLSs. (**a-b**) KEGG and annotation analysis and GSEA functional clustering of differential genes between NETs-treated FLSs and control group.

Supplementary Fig. 3

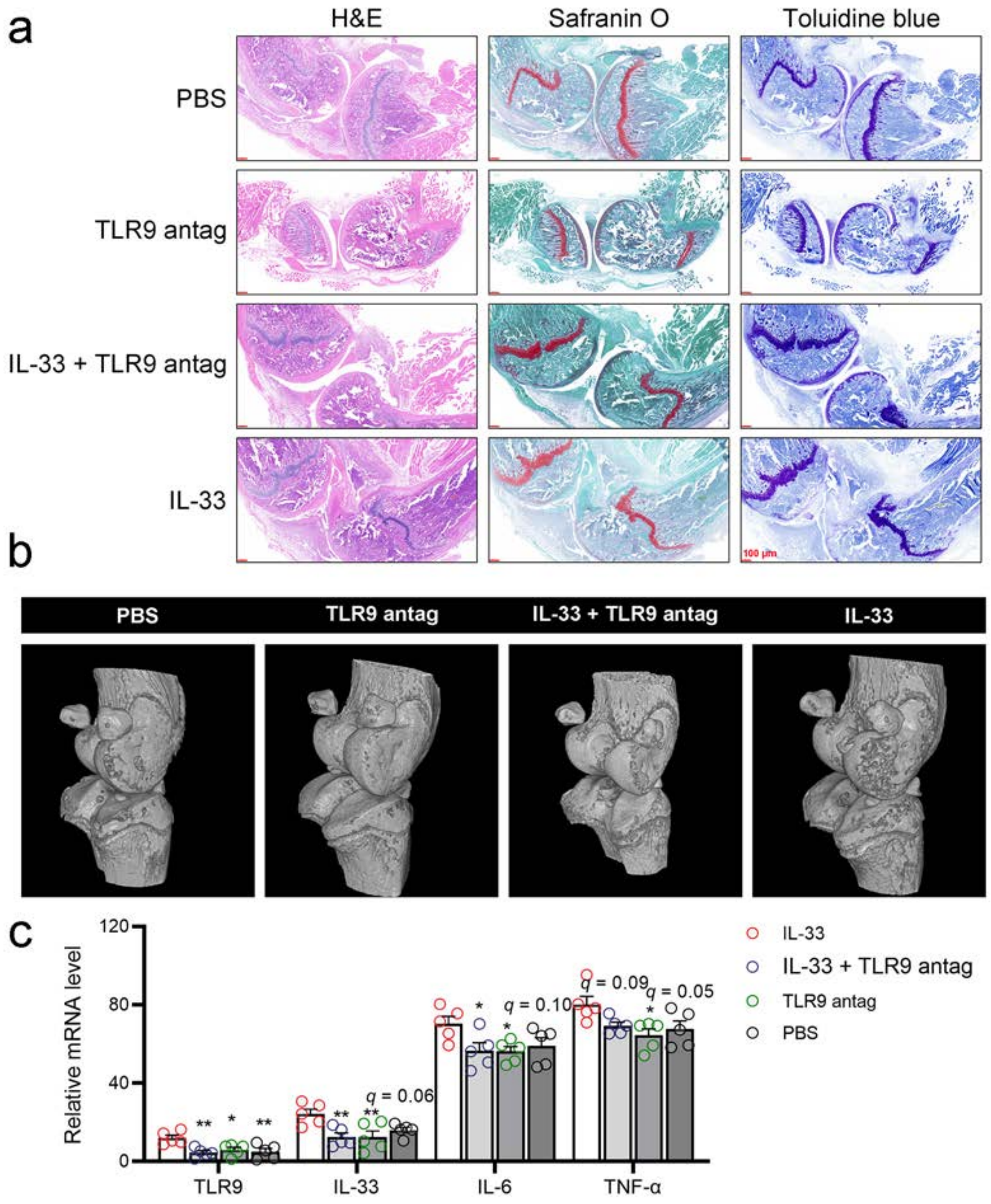

Supplementary Fig. 3. The TLR9 antagonist alleviates IL-33-induced arthritis in CAIA mice. (**a**) Representative images of H&E staining, Safranin O staining, and Toluidine blue staining of joint tissue from IL-33-induced CAIA mice treated with TLR9 antagonist (n = 5). (**b**) Representative micro-CT images of joint tissue from IL-33-induced CAIA mice treated with TLR9 antagonist (n = 5). (**c**) Level of *Tlr9*, *Il33*, *Il6* and *Tnf* mRNA in synovial tissue of IL-33-induced CAIA mice treated with TLR9 antagonist (n = 5).

Supplementary Table 1

Participants' information and clinical characteristics

Characteristics	RA	OA	HD
Number	120	82	98
Synovium/Blood	20/120	20/82	0/98
Age (years)[a]	54.56 ± 12.15	52.23 ± 14.38	48.98 ± 8.94
Sex (Male/Female)	37/83	30/52	24/74
Anti-CCP (IU/mL)[b]	104.0 (32.0, 182.1)	/	/
RF (IU/mL)[b]	99.1 (34.7, 199.0)	/	/
CRP (mm/h)[b]	7.9 (3.1, 20.3)	/	/
ESR (mg/mL)[b]	29.5 (18.0, 43.3)	/	/

[a] Expressed as the means ± SD (standard deviation).

[b] Expressed as the median (25th to 75th percentile).

Abbreviations: RA, rheumatoid arthritis; OA, osteoarthritis; HD, healthy donor; Anti-CCP, anti-cyclic citrullinated peptide antibody; RF, rheumatoid factor; CRP, C-reactive protein; ESR, erythrocyte sedimentation rate.

Supplementary Table 2

Antibodies information used in the study

Antibody	Manufacturer	Catalog
Homo sapiens:		
Anti-Histone H3 (citrulline R2) antibody (1:400 dilution for ELISA, 1:100 dilution for IF, 1:1 000 dilution for WB)	Abcam	ab219407
Anti-Myeloperoxidase antibody (1:500 dilution for ELISA)	Abcam	ab25989
Alexa Fluor 594 goat anti-rabbit IgG (1:2 000 dilution for IF)	Invitrogen	A-11012
ST2 Polyclonal Antibody (0.2 mg/mL for preincubation)	Invitrogen	PA5-47024
Anti-ST2 antibody (1: 100 dilution for IP,1:1 000 dilution for WB)	Abcam	ab317558
Anti-MyD88 antibody (1:1 000 dilution for WB)	Abcam	ab219413
Anti-TRAF6 antibody (1:1 000 dilution for WB)	Abcam	ab137452
Anti-p-Erk1/2 antibody (1:1 000 dilution for WB)	Cell Signaling Technology	4370
Anti-Erk1/2 antibody (1:1 000 dilution for WB)	Cell Signaling Technology	4695
Anti-p-p38 antibody (1:1 000 dilution for WB)	Cell Signaling Technology	4511
Anti-p38 antibody (1:1 000 dilution for WB)	Cell Signaling Technology	8690
Anti-p-IκBα antibody (1:500 dilution for WB)	Beyotime	AF5851
Anti-IκBα antibody (1:500 dilution for WB)	Beyotime	AG2737
Anti-p-NF-κB p65 antibody (1:1 000 dilution for WB)	Cell Signaling Technology	3033
Anti-NF-κB p65 antibody (1:1 000 dilution for WB)	Cell Signaling Technology	8242
HRP-conjugated secondary antibody (1:2 000 dilution for WB)	Beyotime	A0208
IL-33R (ST2) Monoclonal Antibody (3 μL for Flow)	Invitrogen	12-9338-42
Anti-GAPDH antibody (1:1 000 dilution for WB)	Abcam	ab8245
Mus musculus:		
Anti-Myeloperoxidase antibody (1:4 000 dilution for IF)	Abcam	ab208670
Anti-Ly6G antibody (1:1 000 dilution for IF)	servicebio	GB11229
HRP-conjugated secondary antibody (1:5 000 dilution for IF)	Abcam	ab205718

Abbreviations: ELISA, enzyme linked immunosorbent assay; IF, Immunofluorescence; WB, western blot; IP, immunoprecipitations; p-, phosphor-; HRP, Horseradish peroxidase; Flow, Flow cytometry. Dilution or volumes of the antibodies used in the study are shown in parentheses, which recommended by the manufacturer.

Supplementary Table 3

Primers used in the study

Species	Gene Name	Sequence (5′-3′)
Homo sapiens	ST2	Fw：CTGGATATGCGAATGTCACC
		Rv：GCCTGACAATTCTTAAACCAC
	CXCL8	Fw: ACTCCAAACCTTTCCACCCC
		Rv: ATGAATTCTCAGCCCTCTTCAA
	IL33	Fw: TTCGAACTCCAAGATTTCCCC
		Rv: CAAAACTAACAGATTGGTCGTTG
	TLR1	Fw: CTAGCATCTTCCATTTTGCCAT
		Rv: ATGTGCAGACTCTCAGTGTT
	TLR2	Fw: CATCATCAGCCTCTCCAAG
		Rv: TCTAAATGTTCAAGACTGCC
	TLR3	Fw: TTAGCCATGAAGTTGCTGAC
		Rv: AAGGCAAAGGTTTTATCAGA
	TLR4	Fw: GGACTCTGATCCCAGCCATG
		Rv: TAAATCCAGCACCTGCAGT
	TLR5	Fw: GAATTCCTTCCTGCTCCTT
		Rv: GGCCTCCTTGTCAATAGTCA
	TLR6	Fw: GCTTCCATTTTGTTTGCCTT
		Rv: ACTGCAAATTCATTTCCGTC
	TLR7	Fw: GTATTCCCACGAACACCAC
		Rv: CGATAATAACAGTTTTGGCCCAG
	TLR8	Fw: CATTTTCCTGCTAATATCTGG
		Rv: TGTCACATATTTGCCCACC
	TLR9	Fw: CGGAGGCTGGATGTCAG
		Rv: TGTCTTGAGGGCGTTGG
	GAPDH	Fw: GGAGCGAGATCCCTCCAAAAT
		Rv: GGCTGTTGTCATACTTCTCATGG
Mus musculus	*Tlr9*	Fw: CTCCAACCGTATCCACCACC
		Rv: GAGAAGTGCAGGGGGCTAAG
	Il33	Fw: CTCACTGCAGGAAAGTACAGCA
		Rv: TATTTTGCAAGGCGGGACCA
	Il6	Fw: TTCCATCCAGTTGCCTTCTTG
		Rv: GGGAGTGGTATCCTCTGTGAAGTC
	Tnf	Fw: GGACAGTGACCTGGACTGTG
		Rv: GAGGCAACCTGACCACTCTC
	Actb	Fw: TGTCCACCTTCCAGCAGATGT
		Rv: AGCTCAGTA ACAGTCCGCCTAG

Abbreviations: Fw, Forward primer; Rv: Reverse primer.

Supplementary Table 4

Spearman correlation analysis between cytokines and NETs-DNA levels in RA synovial fluid

Cytokines	$citH_3$-DNA		MPO-DNA	
	r	*p*	*r*	*p*
TNF-α	0.0196	0.6818	-0.1744	0.4620
IFN-γ	-0.0887	0.7099	0.1098	0.6450
IL-1β	-0.1880	0.4274	0.0436	0.8551
IL-2	0.1324	0.5780	0.0391	0.8700
IL-2R	-0.2091	0.3763	0.0707	0.7671
IL-4	-0.3679	0.1105	-0.3679	0.1105
IL-5	-0.0211	0.9298	0.0707	0.7672
IL-6	-0.1444	0.5437	0.1068	0.6541
IL-8	0.0271	0.9098	0.0361	0.8799
IL-10	-0.0369	0.8774	0.1121	0.6381
IL-17A	-0.3279	0.1581	0.0602	0.8010
IL-33	0.6103	0.0043**	0.5761	0.0079**

Abbreviations: RA, rheumatoid arthritis; cfDNA, cell free DNA; $citH_3$, citrullinated histone 3; MPO, myeloperoxidase; TNF, tumor necrosis factor; IFN, interferon; IL, interleukin.

Supplementary Table 5

Spearman correlation analysis between IL-33 and other indicators in RA peripheral blood serum

Indicators	IL-33	
	r	*p*
cfDNA	-0.0559	0.7025
citH$_3$-DNA	0.0947	0.5174
MPO-DNA	0.1572	0.2806
sST2	-0.3491	0.0586

Abbreviations: IL, interleukin; RA, rheumatoid arthritis; cfDNA, cell free DNA; citH$_3$, citrullinated histone 3; MPO, myeloperoxidase; sST2, soluble stimulation 2.

Supplementary Table 6

Spearman correlation analysis between DAS28 and other indicators in RA peripheral blood serum

Indicators	DAS28-CRP	
	r	*p*
cfDNA	-0.05215	0.7219
$citH_3$-DNA	0.3074	0.0316*
MPO-DNA	0.5192	0.0001***
IL-33	0.2675	0.0632
sST2	0.4429	0.0142*

Abbreviations: DAS28, disease activity score of 28 joints; RA, rheumatoid arthritis; CRP, C-reactive protein; cfDNA, cell free DNA; $citH_3$, citrullinated histone 3; MPO, myeloperoxidase; sST2, IL, interleukin; soluble stimulation 2.
